# Serum insulin‐like growth factor‐I levels are associated with improved white matter recovery after traumatic brain injury

**DOI:** 10.1002/ana.24971

**Published:** 2017-07-25

**Authors:** Claire Feeney, David J. Sharp, Peter J. Hellyer, Amy E. Jolly, James H. Cole, Gregory Scott, David Baxter, Sagar Jilka, Ewan Ross, Timothy E. Ham, Peter O. Jenkins, Lucia M. Li, Nikos Gorgoraptis, Mark Midwinter, Anthony P. Goldstone

**Affiliations:** ^1^ Computational, Cognitive, and Clinical Neuroimaging Laboratory, Division of Brain Sciences, Imperial College London Hammersmith Hospital London, United Kingdom; ^2^ Imperial Centre for Endocrinology, Imperial College Healthcare NHS Trust St Mary's and Charing Cross Hospitals London, United Kingdom; ^3^ Royal Centre for Defence Medicine, Academic Department of Military Surgery and Trauma Birmingham, United Kingdom; ^4^ Academic Section for Musculoskeletal Disease, Chapel Allerton Hospital University of Leeds Leeds; ^5^ PsychoNeuroEndocrinology Research Group, Neuropsychopharmacology Unit, Centre for Psychiatry, Division of Brain Sciences, Imperial College London Hammersmith Hospital London United Kingdom

## Abstract

**Objective:**

Traumatic brain injury (TBI) is a common disabling condition with limited treatment options. Diffusion tensor imaging measures recovery of axonal injury in white matter (WM) tracts after TBI. Growth hormone deficiency (GHD) after TBI may impair axonal and neuropsychological recovery, and serum insulin‐like growth factor‐I (IGF‐I) may mediate this effect. We conducted a longitudinal study to determine the effects of baseline serum IGF‐I concentrations on WM tract and neuropsychological recovery after TBI.

**Methods:**

Thirty‐nine adults after TBI (84.6% male, median age = 30.5 years, 87.2% moderate–severe, median time since TBI = 16.3 months, n = 4 with GHD) were scanned twice, 13.3 months (range = 12.1–14.9) apart, and 35 healthy controls were scanned once. Symptom and quality of life questionnaires and cognitive assessments were completed at both visits (n = 33). Our main outcome measure was fractional anisotropy (FA), a measure of WM tract integrity, in a priori regions of interest: splenium of corpus callosum (SPCC) and posterior limb of internal capsule (PLIC).

**Results:**

At baseline, FA was reduced in many WM tracts including SPCC and PLIC following TBI compared to controls, indicating axonal injury, with longitudinal increases indicating axonal recovery. There was a significantly greater increase in SPCC FA over time in patients with serum IGF‐I above versus below the median for age. Only the higher IGF‐I group had significant improvements in immediate verbal memory recall over time.

**Interpretation:**

WM recovery and memory improvements after TBI were greater in patients with higher serum IGF‐I at baseline. These findings suggest that the growth hormone/IGF‐I system may be a potential therapeutic target following TBI. Ann Neurol 2017;82:30–43

Traumatic brain injury (TBI) is a common and disabling disease causing severe disability worldwide in 150 to 200 million people annually.^1^ Many more suffer long‐term cognitive and psychological problems with variable natural history.^2^ A major pathological hallmark of TBI is diffuse axonal injury (DAI), resulting from shearing forces sustained during the injury.^3^, ^4^ This is a key determinant of poor clinical outcome after TBI, through the disruption of large‐scale brain network function.^5^ There are currently no drug treatments available to limit axonal injury or promote its recovery.^6^

Diffusion tensor imaging (DTI) provides a sensitive way of quantifying and localizing DAI damage by measuring white matter (WM) and axonal structural integrity, which is difficult using conventional structural magnetic resonance imaging (MRI).^7^ Fractional anisotropy (FA) has been widely used as a measure of WM damage and its recovery.^8^ We and others have demonstrated reductions in FA in many WM tracts after TBI that correlate with cognitive deficits including memory impairment and sustained attention.^9^, ^10^, ^11^, ^12^ Furthermore, changes in FA within the splenium of the corpus callosum (SPCC) and posterior limb of the internal capsule (PLIC) have been shown to predict clinical outcome after TBI.^9^

It is unclear which factors contribute to the wide variation in WM recovery after TBI. One potential factor is post‐traumatic hypopituitarism, which can be produced by TBI and can influence brain repair.^13^, ^14^, ^15^ Studies using gold standard dynamic testing have reported a prevalence of the most common endocrinopathy, growth hormone (GH) deficiency (GHD), of 2.7 to 11.8% in patients >1 year after TBI.^14^, ^16^, ^17^ The prevalence increases with TBI severity, certain TBI mechanisms, and findings on neuroimaging,^18^, ^19^ with a particularly high prevalence of pituitary dysfunction (32.0%) and GHD (15.8%) after moderate–severe blast TBI in soldiers.^20^ The exact diagnostic criteria and use of body mass index (BMI)‐dependent cut‐offs for endocrine tests are, however, important when determining risk factors, especially for GHD.^15^, ^17^, ^20^, ^21^

GH has several targets within the brain.^22^, ^23^, ^24^ Effects are direct or mediated through its downstream hormone insulin‐like growth factor‐I (IGF‐I). Non–TBI‐related GHD is associated with impaired cognitive function, as well as WM tract abnormalities (lower FA) in the corpus callosum and corticospinal tract in children.^25^ GH replacement in this group increases serum IGF‐I and improves attention, memory, and psychological well‐being.^26^, ^27^, ^28^ Variations in serum IGF‐I concentrations have been related to brain structure and cognitive function.^23^ Following stroke, higher serum IGF‐I concentrations at baseline are associated with better neurological and functional outcomes.^29^, ^30^ Mechanistically, GH/IGF‐I deficiency reduces oligodendrocyte turnover in the corpus callosum,^31^ whereas GH/IGF‐I administration increases the formation of new neurons in the hippocampus.^29^, ^32^, ^33^

Following TBI, GH replacement also improves cognition, psychological function, and quality of life (QoL) in patients with GHD.^34^, ^35^, ^36^, ^37^, ^38^ This could be produced through an effect on WM tract recovery, and changes in the levels of IGF‐I might mediate this effect. However, the effects of GHD/IGF‐I status on WM recovery following TBI are unknown.

We hypothesized that IGF‐I status would impact recovery after TBI such that improvement in WM tract FA, and neuropsychological and cognitive measures over time would be greater in those with a higher serum IGF‐I at baseline. MRI including DTI was performed twice along with neuropsychological and cognitive testing in a longitudinal study of patients after TBI, and improvements compared by IGF‐I/GH status.

## Subjects and Methods

### Participants

Thirty‐nine patients with persistent neuropsychological symptoms were recruited in the post‐acute phase following a single TBI (>6 weeks post‐injury). All patients had the following data recorded: age, sex, BMI, mechanism of injury, date of injury, and severity of TBI using Mayo classification.^39^ Research ethics committee approval (Ealing and West London Research Ethics Committee 09/H0707/82) and informed written consent were obtained, in accordance with the Declaration of Helsinki. The following exclusion criteria were applied: (1) diabetes mellitus; (2) pre‐TBI history of psychiatric disorder or neurological disease, including learning disability or attention deficit hyperactivity disorder; (3) current or previous drug abuse or excess alcohol use; and (4) craniotomy following TBI (to avoid image registration difficulties in cases of severe structural changes). All participants had completed endocrine testing prior to enrollment in the study.

Participants had standard structural brain MRI, DTI, and neuropsychological and cognitive assessment at baseline and at follow‐up at least 6 months later. Thirty‐five age‐ and sex‐matched healthy volunteers underwent an identical neuroimaging visit at a single time point as a control group. Baseline scans only from 13 of the patients with TBI had previously been presented in the BIOSAP study, which found greater cognitive dysfunction and WM tract damage in blast TBI soldiers with versus without pituitary dysfunction at baseline.^20^

### Endocrine Assessment

Patients had full endocrine testing at baseline as part of their routine clinical care, as previously described.^20^ Measurements of anterior pituitary hormones (serum IGF‐I, prolactin, thyroid‐stimulating hormone, free thyroxine, luteinizing hormone, follicle‐stimulating hormone, testosterone/estrogen, sex hormone binding globulin) were taken in the late morning/early afternoon in the non‐fasted state. Blood sampling was on average within 1 week of their baseline MRI scan (median = 0.8 weeks, interquartile range [IQR] = 0.3–3.1). Serum IGF‐I was measured by immunoassay using the Immulite 2500 assay (Siemens, Erlangen, Germany), with reference ranges given in Supplementary Table 1. Serum IGF‐I gave stable measurements over time with good reliability on test–retest. Using this assay in n = 195 patients after TBI, the intraclass correlation coefficient of serum IGF‐I was 0.90 (time since TBI: median [IQR] = 17.0 weeks [9.7–45.1], time between IGF‐I measurements = 4.0 weeks [2.4–6.3]).

Subsequently, patients had comprehensive dynamic testing of the GH (and adrenocorticotropic hormone [ACTH]‐cortisol) axis to look for GH (or ACTH) deficiency using endocrine stimulation tests with serial GH measurement after acute administration of glucagon, growth hormone–releasing hormone (GHRH)‐arginine, or insulin‐induced hypoglycemia, as previously described.^35^ Initial screening for GH (and ACTH) deficiency was performed using the glucagon stimulation test with peak GH cutoff <5μg/l.^35^ The diagnosis of GHD was then confirmed using the GHRH‐arginine test, with age‐ and BMI‐adjusted cutoffs,^21^ and/or insulin tolerance test (peak GH <3μg/l), in all patients. Serum GH was assayed using the Immulite 2000 assay. The protocol for diagnosis of other pituitary dysfunction (eg, ACTH or gonadotropin deficiency) was as previously described.^20^ Although limited by the low prevalence, an exploratory subgroup analysis of FA recovery was made for those patients after TBI with (n = 4) versus without (n = 35) confirmed GHD. However, because only 2 subjects with GHD completed neuropsychological and cognitive testing at both time points, these endpoints were not analyzed by GHD status.

### Serum IGF‐I Status

To account for the finding that IGF‐I level declines naturally with age, an age‐adjusted IGF‐I ratio was calculated for each patient. Absolute IGF‐I was divided by the median of the age‐related reference range (see Supplementary Table 1). Patients were then assigned to an “above median‐for‐age” or “below median‐for‐age” IGF‐I group if this ratio was ≥1 or <1, respectively, indicating patients with an absolute IGF‐I in the upper half or lower half of their age‐related reference range.

### Structural Neuroimaging

To assess macrostructural abnormalities, each patient had standard high‐resolution T1 and gradient‐echo T2* MRI on a 3T Achieva scanner (Philips Medical Systems, Best, the Netherlands) using an 8‐channel head coil. Scans were reported by a consultant neuroradiologist with extensive expertise in reporting structural abnormalities following TBI, looking specifically for the number of contusions, presence of microbleeds, DAI, and superficial siderosis. The 4 patients with confirmed GHD also had dedicated gadolinium‐enhanced pituitary MRI scans as part of routine clinical care.

In addition, structural DTI was acquired on each participant to assess directionality of water diffusion as a measure of WM tract integrity through extraction of the primary outcome measure of interest, FA, a measure of unidirectionality (0 = isotropic, 1 = anisotropic; lower number indicates lower unidirectionality and hence greater WM tract damage). Diffusion‐weighted volumes with gradients applied in 16 noncollinear directions were collected in 4 runs, with a total of 64 directions, using the following parameters: 73 contiguous slices, slice thickness = 2mm, field of view = 224mm, matrix = 128 × 128, voxel size = 1.75 × 1.75 × 2mm^3^, *b* value = 1,000s/mm^2^, 4 images with no diffusion weighting (*b*0). DTI images were registered to the *b*0 image by affine transformations to minimize distortion due to motion and eddy currents and then brain‐extracted using the Brain Extraction Tool^40^ from the FMRIB Software Library.^41^, ^42^ The Diffusion Toolbox was used to fit diffusion tensors to the data to generate FA diffusion maps.^43^

### DTI and FA Analysis

**Figure 1:**
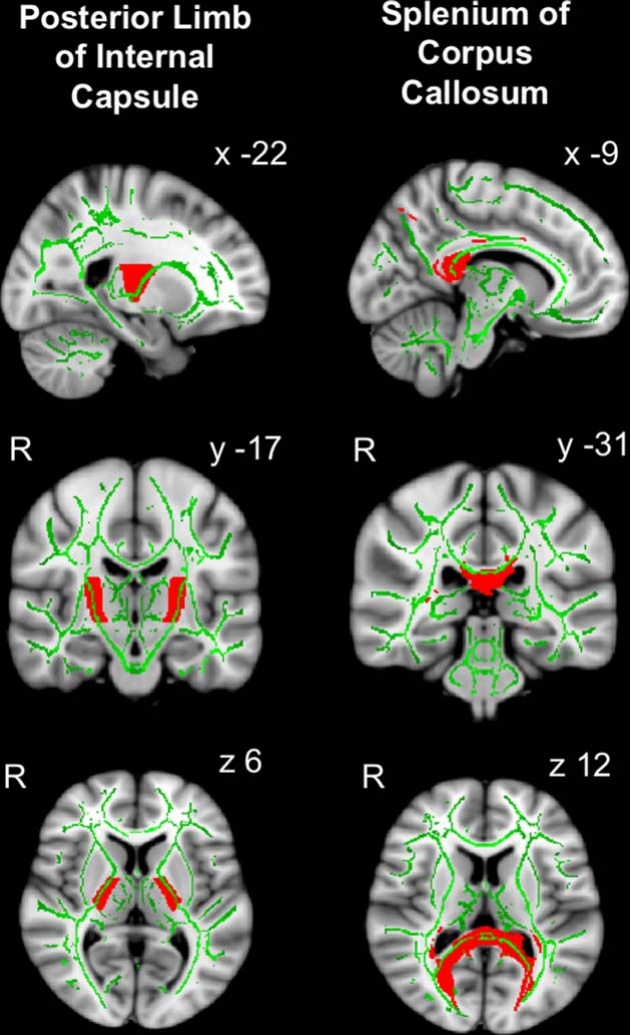
Posterior limb of internal capsule and splenium of corpus callosum white matter (WM) tract regions of interest. Group average fractional anisotropy (FA) skeleton (green, or light gray in print version, threshold > 0.2) from all patients after traumatic brain injury (n = 39) and healthy controls (n = 35) is overlaid onto standard Montreal Neurological Institute 152 (MNI152) 1mm T1 magnetic resonance imaging brain scan. WM tract is defined using the Johns Hopkins University WM tractography atlas with a cutoff threshold of 20% indicated in red (or dark gray in print version). Mean FA values in posterior limb of internal capsule and splenium of corpus callosum WM tract regions of interest were calculated from the average of the voxels indicated by the overlap of the green skeleton and red mask. Coordinates are given in MNI standard space. R = right. [Color figure can be viewed at www.annalsofneurology.org]

FA was analyzed specifically in 2 a priori WM tract regions of interest (ROIs) that have previously been shown to be abnormal after TBI and where normalization of FA predicted clinical outcome, namely the SPCC and the PLIC.^9^ Bilateral ROI masks were defined using the Johns Hopkins University WM tractography atlas with a cutoff threshold >20% (Fig 1), averaging mean FA values in left and right ROIs. FA differences and changes over time were also analyzed across all WM tracts in the whole brain using tract‐based spatial statistics (TBSS) in the FMRIB Software Library with threshold‐free cluster enhancement (TFCE) to correct for multiple comparisons at clusterwise threshold *p* < 0.05. The TBSS steps included: (1) non‐linear alignment of all subjects' FA images into common FMRIB58 FA template space; (2) affine transformation of aligned images into standard Montreal Neurological Institute 152 (MNI152) 1mm space; (3) averaging of aligned FA images to create a 4‐dimensional mean FA image; (4) thinning of the mean FA image to create a mean FA “skeleton” representing the center of all WM tracts, to remove partial‐volume confounds; and (5) thresholding of the FA skeleton at >0.2 to suppress areas of extremely low mean FA and exclude those with considerable interindividual variability.^44^ Nonparametric permutation‐based statistics were employed using randomize and 5,000 permutations.^45^

### FA Comparisons by Group

Analyses and design matrices were constructed to compare FA cross‐sectionally: (1) between patients after TBI (baseline scan 1) versus controls, to examine effects of TBI (ROIs, whole brain); and (2) in patients only between groups at scan 1 only, to examine the effects of GHD and IGF‐I status at baseline only (whole brain). Longitudinal analyses were then performed in patients only: (3) between follow‐up scan 2 versus scan 1, to examine overall recovery over time after TBI (ROIs, whole brain); (4) using an interaction design analysis to examine the effect of GHD and IGF‐I status at baseline and FA recovery over time (ROIs); and (5) using the subtracted FA difference between scan 2 and scan 1, to examine the interaction between GHD and IGF‐I status at baseline and recovery in FA over time (whole brain WM tracts).

### Cognitive and Neuropsychological Assessment

Patients underwent a detailed cognitive testing battery to examine measures previously found to be sensitive to impairments after TBI:^10^, ^20^, ^46^ (1) Wechsler Memory Scale–Third Edition to test immediate and delayed verbal recall (Logical Memory I and II) and working memory (Digit Span); (2) People test from Doors and People test battery to measure associative learning and recall; (3) verbal and letter fluency and color–word (Stroop) tests from the Delis–Kaplan Executive Function System to assess cognitive flexibility, inhibition, and set‐shifting; (4) Trail Making Test to assess executive function; and (5) Wechsler Test of Adult Reading and Wechsler Abbreviated Scale of Intelligence Similarities and Matrix‐Reasoning subsets to assess premorbid verbal and nonverbal reasoning ability at the first visit only.

In addition, patients completed the following symptom, psychological, and QoL questionnaires at both visits that we have used previously in TBI studies: Beck Depression Inventory II (BDI‐II), Epworth Daytime Sleepiness Scale, and the Assessment of GH Deficiency in Adults (AGHDA) and Short Form 36 Health Survey (SF‐36) QoL questionnaires.^20^

### Relationship between DTI FA and Cognitive Findings

To assess potential causative relationships between the influence of IGF‐I status on recovery of FA in WM tracts and cognitive function, significant findings were further examined cross‐sectionally at a single time point in a separate cohort. This consisted of patients after moderate–severe TBI (n = 82) and healthy controls of similar age and sex (n = 40), who underwent similar DTI scanning and an identical neurocognitive assessment. Comparison was made between patients after TBI and controls, and correlations were made between WM FA and cognitive function, both in whole brain WM tract analysis, and in any individual ROIs that were affected by IGF‐I status in the longitudinal study, correcting for age and sex.

DTI scans were performed on a Siemens Verio 3T scanner with diffusion‐weighted volumes with gradients applied in 16 noncollinear directions collected in 4 runs, with a total of 64 directions, using the following parameters: 64 contiguous slices, slice thickness = 2mm, field of view = 256mm, matrix = 128 × 128, voxel size = 2 × 2 × 2mm^3^), repetition time = 9,500 milliseconds, echo time = 103 milliseconds, generalized autocalibrating partially parallel acquisitions (GRAPPA) = 2, *b* value 1,000s/mm^2^, 4 images with no diffusion weighting (*b*0). The DTI processing and analysis pipeline was identical to the longitudinal study.

### Statistical Analyses

Analysis of demographics and ROI FA values was performed using SPSS (v22; IBM, Armonk, NY). Comparison between groups used Mann–Whitney *U* test, chi‐square test (with Yates correction), 2‐way unpaired or paired Student *t* test, and 1‐way analysis of covariance (ANCOVA) or 1‐way repeated measures ANCOVA with post hoc Fisher least significant difference (LSD) test, as appropriate, with statistical threshold *p* < 0.05.

Comparison of FA in ROIs between controls and TBI patients at baseline using ANCOVA included age and sex as covariates. Comparison of FA in ROIs between groups in TBI patients at baseline using ANCOVA included age, sex, TBI severity, and time since TBI as covariates. Repeated measures ANCOVA examined improvement in FA in ROIs, neuropsychological and cognitive function over time in TBI patients, and differences between groups to establish effects of group, time, and group × time interaction without and with addition of the following covariates that might influence recovery: age, sex, TBI severity, time since TBI, and time between DTI scans. Examination of relationships between variables used Pearson or Spearman correlation coefficients *r_P_* or *r_S_*. Time variables were log_10_‐transformed, as they were not normally distributed.

## Results

### Patient Characteristics

Thirty‐three of 39 patients (84.6%) were male, with a median age of 30.5 years (IQR = 24.5–47.0). Thirteen patients (33.3%) had sustained their TBI as a result of a blast exposure from an improvised explosive device, 10 (25.6%) from a road traffic accident, 7 (17.9%) from assault, 6 (15.4%) from a fall, and 3 (7.7%) through a sporting injury. Thirty‐four patients (87.2%) had a moderate–severe TBI by Mayo classification.^39^ The median time between TBI and first scan was 16.3 months (IQR = 3.6–24.5).

Further demographic data and structural abnormalities in the baseline MRI are given for the whole cohort and by IGF‐I group in Table 1 (and by GHD status in Supplementary Table 2). No patients had craniotomy, as these subjects had been excluded from the study.

**Table 1:** Patient Characteristics by IGF‐I Group

Characteristic	All TBI Group	IGF‐I above Age‐Related Median	IGF‐I below Age‐Related Median	*p*, above vs below^a^
No.	39	22	17	
Age at first scan, yr, median (IQR)	30.5 (24.5–47.0)	30.2 (24.3–47.6)	34.5 (25.2–48.8)	0.43
Range	19.6–66.9	19.6–62.2	21.3–66.9	
Male, No. [%]	33 [84.6]	17 [77.3]	16 [94.1]	0.32
Postmenopausal women, No. [%]	3 [7.7]	2 [9.1]	1 [5.9]	0.82
BMI, kg/m^2^, median (IQR)	25.3 (23.1–29.8)	23.6 (19.6–29.0)	25.3 (24.4–30.2)	1.00
Range	17.0–35.0	17.0–32.3	21.3–35.0	
Moderate–severe TBI, No. [%]	34 [87.2]	20 [90.6]	14 [82.4]	0.75
Blast TBI, No. [%]	13 [33.3]	8 [36.3]	5 [29.4]	0.90
Time since TBI, mo, median (IQR)	16.3 (3.6–24.5)	12.5 (4.0–25.9)	17.2 (3.1–33.5)	0.89
Range	1.47–571.0	1.5–571.0	1.5–102.9	
Time between scans, mo, median (IQR)	13.3 (12.1–14.9)	12.4 (10.8–13.4)	14.4 (13.1–15.1)	0.08
Range	6.3–24.6	6.3–24.6	8.9–17.8	
Absolute IGF‐I, nmol/l, median (IQR)^b^	23.8 (19.2–29.5)	28.2 (23.3–35.1)	19.9 (16.8–23.7)	0.02^c^
Range	12.8–74.5	19.2–74.5	12.8–29.0	
Age‐adjusted IGF‐I ratio, median (IQR)	1.1 (0.9–1.2)	1.2 (1.1–1.5)	0.8 (0.8–0.9)	<0.001^c^
Range	0.6–3.0	1.0–2.9	0.6–1.0	
GHD, No. [%]	4 [10.2]	1 [4.5]	3 [17.6]	0.42
Any pituitary deficiency, No. [%]	7 [17.9]	3 [13.6]	4 [23.5]	0.71
Any structural brain abnormality, No. [%]	30 [76.9]	19 [86.4]	11 [64.7]	0.23
≥1 contusion	20 [51.3]	11 [28.2]	9 [52.9]	0.89
No. of contusions, median (IQR)^d^	1 (0–2)	1 (0–1)	1 (0–2)	0.40
Range	0–5	0–2	0–5	
≥1 microbleed, No. [%]	10 [25.6]	8 [36.4]	2 [11.8]	0.17
Superficial siderosis, No. [%]	13 [33.3]	6 [27.3]	7 [41.2]	0.57
Diffuse axonal injury, No. [%]	5 [12.8]	3 [13.6]	2 [11.8]	0.76

IGF‐I groups refer to patients with an absolute IGF‐I concentration above the median‐for‐age or below the median‐for‐age.

a Probability value for comparison of serum IGF‐I group using chi‐square test with Yates correction for categorical variables and Mann–Whitney *U* test for continuous variables.

b To convert nmol/l to ng/ml, divide by 0.131.

c Statistically significant.

d Including those without any contusions.

BMI = body mass index; GHD = growth hormone deficiency; IGF‐I = insulin‐like growth factor‐I; IQR = interquartile range; TBI = traumatic brain injury.

Thirty‐five healthy controls were studied with the following demographics: 23 (65.7%) male (*p* = 0.11 vs TBI) and median age 30.7 = years (IQR = 25.9–38.2, *p* = 0.22 vs TBI).

### GHD and Pituitary Dysfunction

Four (10.2%) patients after TBI had confirmed untreated GHD. Overall, 7 of 39 (17.9%) had pituitary dysfunction (3 isolated GHD, 1 ACTH deficiency, 2 hyperprolactinemia, 1 combined ACTH/GH/gonadotropin deficiencies). Six were soldiers who had sustained blast TBI.^20^ None of these patients had yet started any hormone replacement therapy at the time of their first or second scan. There were no significant differences in age, sex, TBI severity, time since injury, time between scans, prevalence of blast TBI, or prevalence of structural MRI abnormalities at baseline between patients with and without GHD (see Supplementary Table 2). Although dedicated pituitary MRI was only performed in those with GHD, there was no evidence of hypothalamic‐pituitary damage in any patient.

Absolute serum IGF‐I and age‐adjusted IGF‐I ratio tended to be lower (median = 19.0% lower, *p* = 0.11; median = 18.2% lower, *p* = 0.11, respectively) in those patients with versus without GHD (see Supplementary Table 2). Seventy‐five percent of GHD subjects had a serum IGF‐I below the age‐related median.

### IGF‐I Status

Seventeen (43.6%) patients were in the below median‐for‐age IGF‐I group and 22 in the above median‐for‐age IGF‐I group. The below and above median‐for‐age numbers were unequal, as they were defined according to age‐matched reference ranges (see Supplementary Table 1). In the below median‐for‐age IGF‐I group, 1 of 17 (5.6%) had a serum IGF‐I < 5th percentile (age‐adjusted normal range); in the above median‐for‐age IGF‐I group, 4 of 22 (18.2%) had a serum IGF‐I > 95th percentile. There were no significant differences in age, sex, TBI severity, time since injury, BMI, or prevalence of blast TBI (below vs above median‐for‐age IGF‐I group = 29.4% vs 36.3%, *p* = 0.90), structural MRI abnormalities at baseline, or post‐menopausal females between IGF‐I groups (see Table 1). There was a trend toward a greater length of time between the two scans in the below median IGF‐I group compared to the above median IGF‐I group (median = 14.4 vs 12.5 months, *p* = 0.08).

Only 3 of 17 (17.6%) patients in the below median‐for‐age IGF‐I group had GHD confirmed on dynamic testing, compared to 1 of 22 (4.5%) in the above median‐for‐age IGF‐I group (*p* = 0.15). Of the 4 patients with confirmed GHD, none had serum IGF‐I < 5th percentile, 3 had IGF‐I between 5th percentile and median, and 1 had IGF‐I between median and 95th percentile.

### Effect of TBI on WM tracts

#### TBI Patients Show Evidence of Axonal Injury

**Figure 2:**
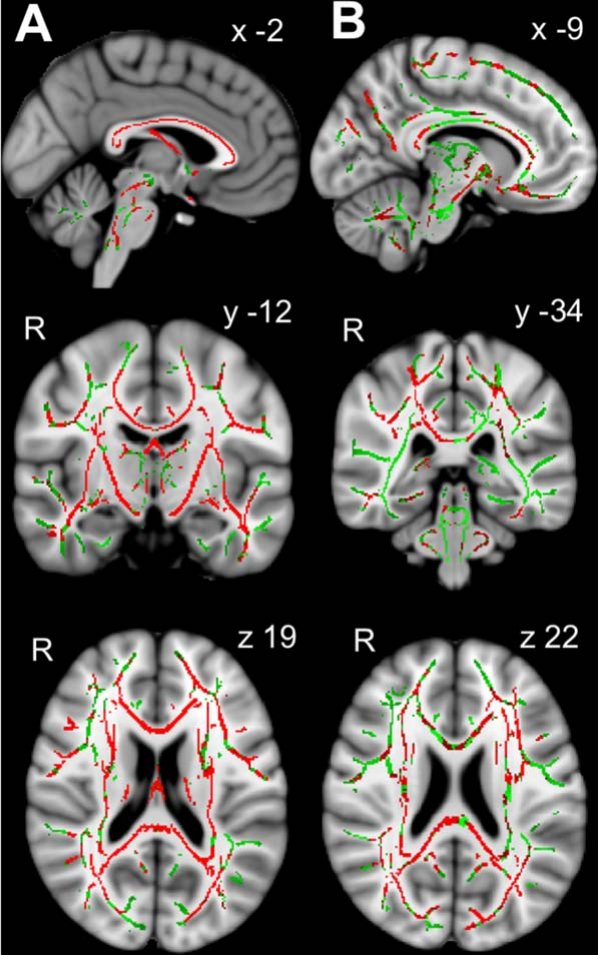
White matter tract fractional anisotropy (FA) in patients after traumatic brain injury (TBI). (A) Lower white matter tract FA in patients after TBI. Group average statistical maps show voxels displaying lower FA in patients with TBI (n = 39) compared to healthy controls (n = 35) displayed in red, or dark gray in print version (threshold‐free cluster enhancement [TFCE] clusterwise threshold *p* < 0.05, correcting for age and sex), overlaid on combined whole group FA skeleton (green, or light gray in print version), displayed on standard Montreal Neurological Institute 125 (MNI125) 1mm T1 magnetic resonance imaging (MRI) brain scan. Coordinates given in MNI standard space. (B) White matter tract FA improvement between scans. Group average statistical maps show voxels displaying higher FA in TBI patients at follow‐up scan (in red, or dark gray in print version) compared to baseline scan overlaid on combined whole group FA skeleton (green, or light gray in print version) using TFCE clusterwise threshold *p* < 0.05, correcting for age, time since injury, sex, TBI severity, and time between scans. Results are displayed on standard MNI125 1mm T1 MRI brain scan. Coordinates are given in MNI standard space. R = right. [Color figure can be viewed at www.annalsofneurology.org]

**Figure 3:**
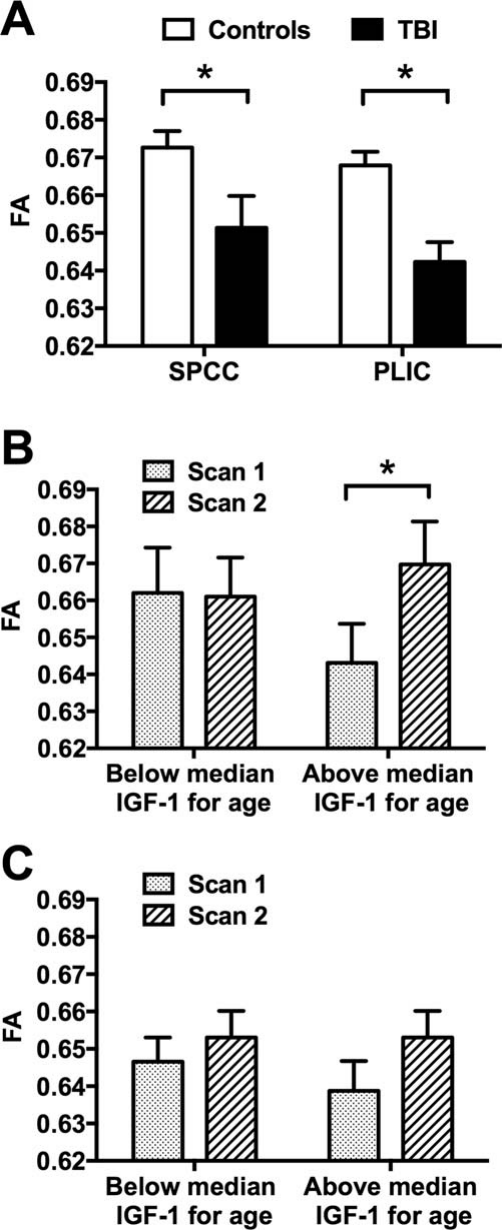
White matter tract fractional anisotropy (FA) in regions of interest (ROIs) in patients after traumatic brain injury (TBI) at baseline and recovery over time by insulin‐like growth factor‐I (IGF‐I) group. (A) Lower FA in splenium of corpus callosum (SPCC) and posterior limb of internal capsule (PLIC) ROIs in patients after TBI *(black bars)* compared to controls *(white bars)* at baseline scan, adjusting for age and sex (*p < 0.05, analysis of covariance [ANCOVA] with post hoc Fisher least significant difference [LSD] test). (A, B) Greater improvement in FA in SPCC (B), but no difference in improvement in FA in PLIC (C), between scan 1 *(dotted bars)* and scan 2 *(striped bars)* in the above median‐for‐age IGF‐I group compared to the below median‐for‐age IGF‐I group, adjusting for age, sex, TBI severity, time since TBI, and time between scans (*p < 0.05, repeated measures ANCOVA with post hoc Fisher LSD test). Data are given as mean ± standard error of the mean. Statistics for repeated measures ANCOVA: (B) effect of time: *F*_1,32_ = 0.15, *p* = 0.70; group: *F*_1,32_ = 0.12, *p* = 0.74; time × group interaction: *F*_1,32_ = 5.72, *p* = 0.023; (C) effect of time: *F*_1,32_ = 0.34, *p* = 0.56; group: *F*_1,32_ = 1.01, *p* = 0.32; time × group interaction: *F*_1,32_ = 1.31, *p* = 0.26.

In whole brain analysis, FA was significantly lower in many WM tracts including splenium, body and genu of corpus callosum, PLIC, cerebellar peduncles, anterior region of the corona radiata, and internal capsule in patients following TBI at baseline scan 1 (n = 39) compared to healthy controls (n = 35; Fig 2A). As expected, compared to the healthy control group, the TBI group overall had significantly lower FA in the a priori ROIs SPCC (effect size mean ± standard error of the mean = −0.021 ± 0.009, 95% confidence interval [CI] = −0.003 to −0.040, *t*_72_ = −2.26, *p* = 0.027) and PLIC (effect size = −0.026 ± 0.006, 95% CI = −0.039 to −0.013, *t*_72_ = −3.90, *p* < 0.001; Fig 3).

### WM Tract Recovery over Time

#### INCREASED FA OVER TIME SUGGESTS AXONAL RECOVERY FOLLOWING TBI

Across the whole TBI group, FA increased from scan 1 to follow‐up scan 2 in a number of WM tracts including splenium, body, and genu of corpus callosum, subcortical regions, right frontal region, and widespread ventral regions (see Fig 2B). This suggests an overall recovery of axonal injury in these tracts. This was also observed in the a priori ROIs SPCC (effect size = 0.015 ± 0.007, 95% CI = 0.001–0.281, *t*_38_ = 2.2, *p* = 0.035) and PLIC (effect size = 0.016 ± 0.004, 95% CI = 0.003–0.020, *t*_38_ = 3.1, *p* = 0.005). There were no significant effects of age (*p* = 0.35–0.51), sex (*p* = 0.14–0.60), TBI severity (*p* = 0.11–0.23), time since TBI (*p* = 0.33–0.73), or time between scans (*p* = 0.58–0.90) on the change in FA over time (covariate × time interaction), when including each factor separately as a within‐subject covariate in a repeated measures ANCOVA model.

### Effect of IGF‐I Group on WM Tract FA

#### No Cross‐Sectional Influence of IGF‐I Group on FA at Baseline

Within the TBI group, there was no significant effect of IGF‐I group on FA at baseline in whole brain analysis, with or without inclusion of covariates. This lack of any effect was determined by the absence of any voxels within any WM tract reaching the clusterwise TFCE threshold *p* < 0.05.

#### Influence of IGF‐I Group on Recovery in FA over Time

Within the TBI group, there was a significant IGF‐I group × time interaction (*F*_1,37_ = 4.62 *p* = 0.038). This resulted from a significant increase in FA in the a priori SPCC ROI over time in the above median‐for‐age but not in the below median‐for‐age IGF‐I group (above median‐for‐age IGF‐I group effect size = +0.027 ± 0.008, 95% CI = 0.009–0.044, *F*_1,37_ = 9.82, *p* = 0.03; below median‐for‐age IGF‐I group effect size = −0.001 ± 0.100, 95% CI = −0.021 to 0.190, *F*_1,37_ = 0.01, *p* = 0.92). Similar results were seen when including covariates in the repeated measures ANCOVA (IGF‐I group × time interaction: *F*_1,32_ = 5.66, *p* = 0.023; above median‐for‐age IGF‐I group effect size = +0.030 ± 0.009, 95% CI = 0.011–0.048, *F*_1,32_ = 10.60, *p* = 0.003; below median‐for‐age IGF‐I group effect size = −0.011 ± 0.010, 95% CI = −0.026 to 0.017, *F*_1,32_ = 0.21, *p* = 0.65; see Fig 3B). There was no significant difference in SPCC FA between IGF‐I groups at baseline. A similar interaction for FA improvement in SPCC over time was also seen when including blast versus nonblast TBI in the analysis of co‐variance model (IGF‐I group × time interaction: *F*_1,36_ = 4.27, *p* = 0.046), with no significant effect of blast TBI overall (*F*_1,36_ = 0.28, *p* = 0.60) or blast TBI × time interaction (*F*_1,36_ = 1.94, *p* = 0.17).

A similar interaction was seen for FA improvement in SPCC over time when excluding the 4 subjects without moderate–severe TBI (IGF‐I group × time interaction: *F*_1,29_ = 3.74, *p* = 0.063), or when excluding 2 subjects with a prolonged time since TBI > 100 months (IGF‐I group × time interaction: *F*_1,31_ = 5.06, *p* = 0.032, including age, sex, severity, and time between scans).

There was, however, no significant effect of IGF‐I adjusted for age as a continuous rather than categorical variable on FA recovery in the SPCC (time × IGF‐I age‐adjusted ratio interaction: *F*_1,37_ = 0.25, *p* = 0.62; Spearman correlation coefficient for delta SPCC FA: *r* = +0.20, *p* = 0.22).

There was no significant effect of IGF‐I group on increase in FA over time in the a priori PLIC ROI either without any covariates (IGF‐I group × time interaction: *F*_1,37_ = 0.57, *p* = 0.46) or when including covariates (IGF‐I group × time interaction: *F*_1,32_ = 1.31, *p* = 0.26; see Fig 3C).

In whole brain subtraction analysis, there was no significant effect of IGF‐I group on the change in FA between scans 2 and 1, with or without inclusion of covariates. A similar lack of effect of IGF‐I group was also seen when restricting the skeleton in whole brain analysis to only those voxels showing a significant increase in FA between scans (see Fig 2B).

### Effect of IGF‐I Group on Neuropsychological and Cognitive Variables

**Figure 4:**
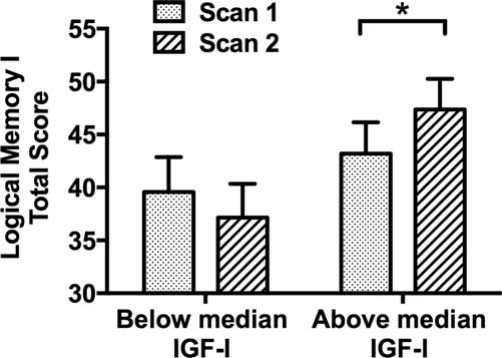
Recall memory in patients after traumatic brain injury (TBI) at baseline and recovery over time by insulinlike growth factor‐I (IGF‐I) group. There was greater improvement in Logical Memory I (LMI) total score (immediate recall) in the above median‐for‐age IGF‐I group compared to the below median‐for‐age IGF‐I group, adjusting for age, sex, TBI severity, time since TBI, and time between scans (*p < 0.05, repeated measures analysis of covariance [ANCOVA] with post hoc Fisher least significant difference test). There was a significant increase in LMI score between scan 1 *(dotted bars)* and scan 2 *(striped bars)* for the IGF‐I group above but not below median at baseline, and no significant difference between IGF‐I groups at baseline. Data are given as mean ± standard error of the mean. Statistics for repeated measures ANCOVA: effect of time: *F*_1,26_ = 1.33, *p* = 0.26; group: *F*_1,26_ = 2.56, *p* = 0.12; time × group interaction: *F*_1,26_ = 4.38, *p* = 0.046.

Within the TBI group, there was a significant IGF‐I group × time interaction (*F*_1,26_ = 4.38, *p* = 0.046) in the Logical Memory I total score (measure of immediate recall). This resulted from a significant increase in memory over time in the above median‐for‐age but not the below median‐for‐age IGF‐I group (above median‐for‐age IGF‐I group effect size = +4.19 ± 2.01, 95% CI = 0.06–8.31, *F*_1,26_ = 4.34, *p* = 0.047; below median‐for‐age IGF‐I group effect size = −2.42 ± 2.22, 95% CI = −0.699 to 2.15, *F*_1,26_ = 1.19, *p* = 0.29) when including covariates (age, sex, severity, time since injury, and time between scans) in the repeated measures ANCOVA, despite scores being similar between groups at baseline (*F*_1,26_ = 0.60, *p* = 0.45; Fig 4).

There was no significant correlation between the improvement in SPCC FA over time and the improvement in Logical Memory I total score over time in patients after TBI, either alone (*r_P_* = +0.20, *p* = 0.28) or when correcting for age, sex, severity, time since injury, and time between scans (partial *r_P_* = +0.26, *p* = 0.18).

There was no significant IGF‐I group × time interactions or effects of time for any of the other cognitive measures, or symptom (BDI‐II, Epworth Daytime Sleepiness Scale) or QoL (SF‐36, AGHDA) questionnaire scores (Supplementary Table 3). There were, however, significant improvements over time in associative memory (People Test; *F*_1,32_ = 6.12, *p* = 0.019) and cognitive flexibility (color–word Stroop; *F*_1,29_ = 5.67, *p* = 0.024), independent of IGF‐I group.

### Relationship between DTI FA and Neurocognitive Findings in Cross‐Sectional Cohort

#### Lower SPCC FA and Worse Memory in Patients after TBI and Controls in Separate Cohort

**Figure 5:**
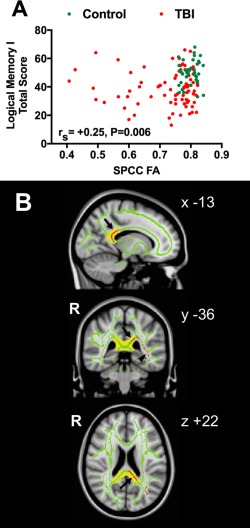
Relationship between recall memory and splenium fractional anisotropy (FA) in patients after traumatic brain injury (TBI) and controls. (A) Relationship between mean splenium of corpus callosum (SPCC) FA as independent variable and Logical Memory I (LMI) total score (immediate recall) as dependent variable, in patients after moderate–severe TBI (n = 82, *red circles*) and controls of similar age and sex (n = 40, *green circles*), without adjustment for any covariates. *r_S_* represents Spearman correlation coefficient for combined groups. This represents a separate cross‐sectional cohort of subjects to the longitudinal cohort. (B) Group average statistical maps showing voxels (in red) in SPCC *(arrows)* and inferior longitudinal fasciculus *(arrowhead)* white matter (WM) tracts whose FA is positively correlated with LMI total score (immediate recall) in TBI patients (n = 82). Results are overlaid on combined group FA skeleton (green, threshold > 0.2), using threshold‐free cluster enhancement clusterwise threshold *p* < 0.05, correcting for age and sex. Results are displayed on standard Montreal Neurological Institute 125 (MNI125) 1mm T1 magnetic resonance imaging brain scan. SPCC WM tract defined using the Johns Hopkins University WM tractography atlas with a cutoff threshold of 20% is indicated in yellow. Mean FA values in the SPCC WM tract region of interest in A were calculated from the average of the voxels indicated by the overlap of the green skeleton and yellow mask. Coordinates are given in MNI standard space. R = right.

Both SPCC FA and Logical Memory I total score (measure of immediate recall) were significantly lower in a separate cross‐sectional cohort of patients after moderate–severe TBI (n = 82) than in controls of similar age and sex (n = 40; Table 2, Fig 5A).

**Table 2:** Comparison between Splenium FA and Immediate Memory Recall in Separate Cross‐Sectional Cohort of Controls and Patients after TBI

	Controls	TBI	Statistic	*p*
No.	40	82		
Age, yr, mean ± SD (range)	42.1 ± 12.6 (21–72)	42.4 ± 12.0 (20–72)	*t* −0.14	0.89
Male, No. [%]^a^	30 [75.0]	69 [84.1]	χ^2^ 0.93	0.33
Time since TBI, median mo, {IQR}	n/a	30.5 {12.5–153.8}	n/a	n/a
SPCC FA, median {IQR} (range)^b^	0.8037 {0.7760, 0.8203} (0.7603–0.8408)	0.7667 {0.6989, 0.7926} (0.4072–0.8221)	*Z* −5.13	<0.001
Logical Memory I total, mean ± SD (range)	50.00 ± 8.39 (33–68)	39.24 ± 12.15 (13–66)	*t* 5.70	<0.001

Comparison of demographics, average FA in SPCC, and Logical Memory I total score (immediate recall) in separate cross‐sectional cohort of healthy controls and patients after moderate–severe traumatic brain injury.

Comparison between groups was made by 2‐tailed unpaired Student *t* test, except as indicated:

a Chi‐square test.

b Mann–Whitney test, as data were not normally distributed.

FA = fractional anisotropy; IQR = interquartile range; n/a = not applicable; SD = standard deviation; SPCC = splenium of the corpus callosum; TBI = traumatic brain injury.

#### Correlation between Lower SPCC FA and Worse Memory in Patients after TBI and Controls

Furthermore, SPCC FA (average in whole ROI) was positively correlated with Logical Memory I total score in patients after TBI and controls (*r_S_* = +0.25, *p* = 0.006; see Fig 5A), and when adjusting for age and sex (*r_S_* = +0.23, *p* = 0.012).

In whole brain WM tract analysis, a positive correlation between Logical Memory I total score in patients after TBI and FA was seen only in the SPCC and the left inferior longitudinal fasciculus, using cluster‐corrected TFCE *p* < 0.05, including age and sex as covariates (see Fig 5B).

### Effect of GHD on WM Tract FA

Only 4 of 39 subjects had confirmed GHD. However, we performed exploratory investigations to determine whether there was an effect of GHD on WM recovery despite the underpowered nature of such analyses.

#### No Cross‐Sectional Influence of GHD on FA at Baseline

Within the TBI group, there was no significant effect of GHD on FA at baseline in whole brain WM tract analysis, with or without inclusion of covariates (age, sex, TBI severity, time since TBI). This lack of any effect was determined by the absence of any voxels within any WM tract reaching the clusterwise TFCE threshold *p* < 0.05.

#### No Longitudinal Influence of GHD Status on Change in FA over Time

**Figure 6:**
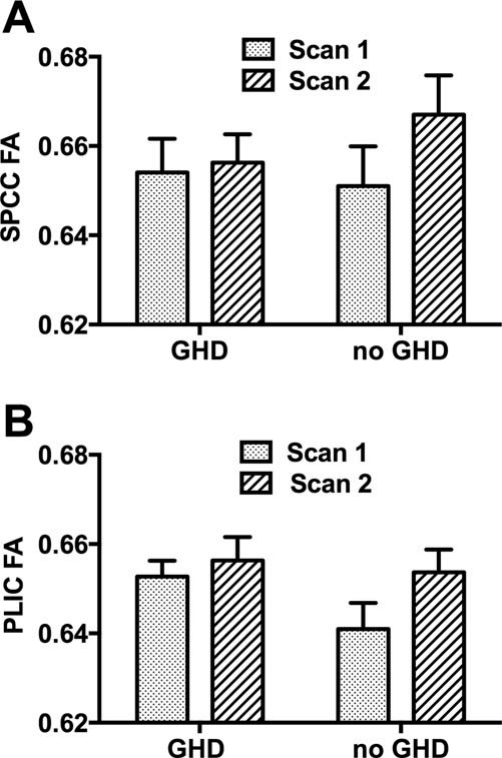
White matter tract fractional anisotropy (FA) in regions of interest in patients after traumatic brain injury (TBI) at baseline and recovery over time by growth hormone deficiency (GHD) status. There was no significant difference in improvement in FA in either (A) splenium of corpus callosum (SPCC) or (B) posterior limb of internal capsule (PLIC) between scan 1 *(dotted bars)* and scan 2 *(striped bars)* in patients after TBI between those without (n = 35) and with (n = 4) GHD, adjusting for age, gender, TBI severity, time since injury, and time between scans. Statistics for repeated measures analysis of covariance: (A) effect of time: *F*_1,32_ = 0.24, *p* = 0.63; group: *F*_1,32_ = 2.23, *p* = 0.15; time × group interaction: *F*_1,32_ = 1.55, *p* = 0.22; (B) effect of time: *F*_1,32_ = 0.44, *p* = 0.51; group: *F*_1,32_ = 0.09, *p* = 0.77; time × group interaction: *F*_1,32_ = 0.58, *p* = 0.45.

Within the TBI group, there was no significant effect of GHD on the increase in FA over time in either the SPCC or PLIC ROIs (GHD status × time interaction: *p* = 0.29–0.45). This remained non‐significant with the inclusion of the following covariates: age, sex, TBI severity, time since TBI, and time between DTI scans (GHD status × time interaction: *p* = 0.22–0.45; Fig 6).

Similarly, in whole brain WM tract subtraction analysis, there was no significant effect of GHD on the change in FA between scans 2 and 1, with or without inclusion of covariates. A similar lack of effect of GHD was also seen when restricting the skeleton in whole brain WM tract analysis to only those voxels showing a significant increase in FA between scans (see Fig 2B).

## Discussion

Our results show that recovery of diffuse axonal injury after TBI, measured using DTI, appears to be influenced by serum IGF‐I concentration at baseline. We focused on the corpus callosum, as this region is frequently damage by TBI and its recovery has been shown to predict clinical outcome.^9^ A greater increase in FA over time in the corpus callosum, indicative of WM recovery, was seen in patients who had serum IGF‐I above the median for their age, compared to those with IGF‐I below median. We also found that immediate memory recall improved to a greater extent in those with serum IGF‐I above the median for their age compared to those below. The association of IGF‐I with improved WM and memory recovery after TBI requires further exploration, but if a direct neuroendocrine effect on brain recovery is confirmed, this may have important therapeutic implications.

Human serum IGF‐I, a 70‐amino‐acid polypeptide, is thought to mediate many of the trophic effects of GH through both hepatic and local production. The effects of both systemic IGF‐I and GH on the brain are not completely clear or disentangled; however, both peptides are able to penetrate the blood–brain barrier, and peripheral IGF‐I and GH administration can improve spatial learning and memory in animal studies, suggesting that similar pathways mediate these effects.^23^, ^47^

At a mechanistic level, GH receptors are ubiquitous throughout the brain and GH/IGF‐I can have a direct neurogenic effect on proliferation of neurons, oligodendrocytes, and new blood vessels.^32^ Deficiency of either hormone can also decrease neuronal survival time, suggestive of a neuroprotective effect.^47^ GH also influences the hippocampal N‐methyl‐D‐aspartate receptor system with effects on memory acquisition. GH/IGF‐I may also promote brain repair through the local induction and release of a number of neurotrophic factors such as vascular endothelial growth factor, epidermal growth factor, and brain‐derived neurotrophic factor.^48^ Other suggested mechanisms are directly through reduction of neuroinflammation or improvement in peripheral metabolic factors such as metabolic syndrome.

GHD following TBI is a well‐recognized post‐traumatic phenomenon but appears not be as common as initially reported. More recent larger studies have reported a prevalence of GHD of 2.7 to 11.8% of 112 patients in a Dutch cohort (using insulin tolerance test, or GHRH‐arginine test if contraindicated),^16^ and 4.5 to 11.8% of 439 patients in a Danish cohort (using insulin tolerance test or GHRH‐arginine/GHRH‐pyridostigmine test),^17^ both including patients >1 year after TBI. In the Imperial TBI clinic, we have found a prevalence of GHD of 7.4% of 176 patients after TBI of mixed severity (median = 0.47 years since TBI), and 8.5% of 258 patients after moderate–severe TBI only, with diagnosis requiring inadequate GH response to both glucagon, and GHRH‐arginine testing or insulin‐induced hypoglycemia.^20^ In this study, we found only 4 of 39 patients (10.3%) had GHD using these criteria, a prevalence enhanced by one‐third of patients having blast TBI, which is associated with a particularly high risk of pituitary dysfunction.^20^ GHD at baseline did not influence increase in FA in either ROI, although these results should be interpreted with caution, because only 4 patients had GHD in this cohort, meaning that we are likely to have significant type II errors in examining the effects of WM tract recovery.

We therefore chose to primarily study serum IGF‐I as a biomarker given the potential convergence of the GH/IGF‐I axis on brain recovery. Serum IGF‐I is the biomarker used to titrate GH replacement therapy in subjects who have GHD, with a target IGF‐I in the upper half of the age‐related reference range being the usual target for dose adjustment.^49^ We were not powered to look for effects of extremely low or high serum IGF‐I concentrations, because only 5 of 39 patients (12.8%) had serum IGF‐I outside the 95% CI for their age (1 above, 4 below).

Another limitation of our study is that we did not measure serum IGF‐binding proteins that may alter free IGF‐I concentrations, although none of our patients was malnourished, and there were no significant differences in other factors that might influence IGF‐I concentrations such as BMI and post‐menopausal status between IGF‐I groups.

Finally, we had included 4 patients with mild TBI in our cohort, and 2 with a very long time from injury (>100 months). In these patients, we may expect that the dynamic WM changes over time may be reduced and therefore could have influenced our overall result. However, in 2 separate analyses excluding these patients, the effect of IGF‐I on WM recovery remained similar. Furthermore, there was a high prevalence of blast TBI in our cohort. However, there was no difference in the prevalence of blast TBI between IGF‐I groups, and inclusion of blast TBI category did not influence the finding of greater FA recovery in SPCC over time in the above median‐for‐age IGF‐I group. This makes it unlikely that the results were influenced by the inclusion of many subjects with blast TBI.

We chose to focus on FA as a measure of WM tract recovery, as this is a reliable measure of WM integrity.^10^, ^11^ Given that DTI measures water diffusion in WM tracts and FA is a measure of diffusion directionality, we cannot be certain that this specifically relates to underlying WM damage and repair in these patients. However, we do show a striking reduction in FA across the whole brain in patients after TBI compared to controls and that FA increases over time after TBI.

Although interpretation is limited by the relatively small sample size and multiple neuropsychological and cognitive tests used, we also found that only the group with IGF‐I above the median for age had a significant improvement in immediate memory recall over time. We have previously shown that after TBI, FA measurements in particular WM tracts relate to multiple neuropsychological domains.^10^, ^11^, ^46^ In a separate cross‐sectional cohort of patients after moderate–severe TBI and controls who were scanned once, we confirmed both lower SPCC FA and lower immediate verbal memory recall in patients after TBI compared to controls. Furthermore, SPCC FA was positively correlated with immediate verbal memory recall.

This association may reflect a direct role of the SPCC in immediate verbal memory. The SPCC contains fibers from the ventral, retrosplenial, subsplenial, and dorsoposterior cingulate cortex (Brodmann areas [BA] 23, 29–31), in addition to sensory and visual association cortices (BA 5, 7, 18, 19).^50^ Diffusivity measures in the SPCC, indicating greater WM tract damage, have been negatively correlated with functional connectivity of the posterior cingulate cortex (PCC) after TBI.^51^ Infarction of the SPCC and retrosplenial region can lead to verbal memory problems.^52^, ^53^, ^54^ In patients with multiple sclerosis, verbal learning performance was inversely associated with lesions in the SPCC, lower FA in SPCC predicted a greater progression of disability, and lower FA in the corpus callosum predicted worse verbal memory, longitudinally over 5 years.^55^, ^56^

Alternatively, the SPCC‐memory results may be a marker of related damage to nearby WM tracts, or overall severity of TBI and associated WM damage, with the larger size and higher FA of the SPCC providing greater sensitivity to detect damage. Damage to the neighboring cingulum bundle, the WM tract connecting the parahippocampal gyrus to the PCC, has been associated structurally and functionally with the development of acute post‐traumatic amnesia after TBI, where there is pronounced inability to encode new memories.^57^

However, in the longitudinal study, we could not demonstrate any correlation of the improvement in SPCC FA with improvement in immediate verbal memory recall, nor any associated change in any other cognitive, symptom, or QoL measures, which may reflect the smaller numbers. Future larger studies will need to confirm these preliminary findings, see whether they are also found in other WM tracts, and address whether any greater increase in FA seen over time in those with higher IGF‐I concentrations is also associated with greater improvements in other neuropsychological functions, and whether other neuroimaging markers such as brain atrophy are also influenced by IGF‐I status.

Our ROI analysis was restricted to areas expected to see a change in FA based on previous studies.^9^, ^58^ Whole brain WM tract analyses using TBSS showed a striking effect of time on FA recovery across many WM tracts. However, we did not find any significant interaction with IGF‐I group. This may be due to several factors, including small numbers given the need for robust statistical correction for multiple comparisons in whole brain analysis, and variability in the time since TBI and time between scans in our cohort, although these did not appear to be important factors influencing FA recovery in ROI analysis. Future studies should utilize larger cohorts with more consistent time periods. Unlike the categorical classification, we did not see any influence of serum IGF‐I as a continuous variable on SPCC FA recovery. However, this assumes that there is a straightforward relationship between IGF‐I status and WM tract recovery and there may be a cutoff level on the IGF‐I dose–response curve. Measurement of cerebrospinal fluid IGF‐I and serum IGF‐I at serial time points would also be helpful in future studies.

In conclusion, we have presented preliminary work demonstrating that higher concentrations of serum IGF‐I than median for age in adults following a TBI are associated with higher WM recovery in the SPCC, and greater improvements in immediate verbal memory over time, irrespective of the presence of GHD. Furthermore, in a larger expanded cross‐sectional cohort, greater SPCC WM tract damage was correlated with worse immediate verbal memory in patients after TBI, consistent with a possible direct relationship. There are limited treatments to improve recovery from TBI, and relatively little is known about what predicts the prognosis for patients. Future larger studies are needed to confirm and extend these findings. If confirmed, this reveals the possibility that it may be worthwhile to investigate whether GH treatment to raise serum IGF‐I into the upper part of the age‐related reference range might aid recovery following TBI, regardless of whether GHD is present using dynamic tests.

## Author Contributions

Study concept and design: C.F., D.J.S., M.M., A.P.G.; data acquisition and analysis: C.F., D.J.S., P.J.H., A.E.J., J.H.C., G.S., D.B., S.J., E.R., T.E.H., P.O.J., L.M.L., N.G., A.P.G.; drafting the manuscript and figures: C.F., D.J.S., A.P.G.; review and editing the manuscript: all authors.

## Potential Conflicts of Interest

Nothing to report.

## Supporting information

Additional supporting information can be found in the online version of this article.

Supporting Information
