# Supplementary material for: Serum insulin‐like growth factor‐I levels are associated with improved white matter recovery after traumatic brain injury
Source: Ann Neurol. 2017 Jul 25;82(1):30–43. doi: 10.1002/ana.24971 (PMC5601275; doi:10.1002/ana.24971)
Supplement: Supplementary file 1 — Supporting Information [file ANA-82-30-s001.pdf]

SUPPLEMENTARY INFORMATION

Serum IGF-I levels are associated with improved white matter recovery after TBI

Feeney C, Sharp DJ, Hellyer PJ, Jolly AE, Cole JH, Scott G, Baxter D, Jilka S, Ross E, Ham TE, Jenkins PO, Li LM, Gorgoraptis N, Midwinter M, Goldstone AP

SUPPLEMENTARY TABLES

Supplementary Table 1. Age-related reference ranges for serum IGF-I assay

Supplementary Table 2. Patient characteristics by GH deficiency status

Supplementary Table 3. Neuropsychological outcome measures in patients after TBI at baseline and recovery over time by IGF-I status.

Supplementary Table 1. Age-related reference ranges for serum IGF-I assay

Age<br>yr	IGF-I median<br>nmol/L	IGF-I lower<br>nmol/L	IGF-I upper<br>nmol/L
18	40.0	21.2	75.9
19	33.9	18.3	62.8
20	30.2	16.5	55.1
21-25	26.4	15.1	46.5
26-30	25.5	15.2	42.8
31-35	24.4	15.0	39.9
36-40	22.9	14.2	36.9
41-45	21.3	13.1	34.7
46-50	20.0	12.2	32.8
51-55	18.7	11.3	30.9
56-60	17.6	10.5	29.3
61-65	16.4	9.8	27.6
66-70	15.3	9.0	26.0
71-75	14.3	8.3	24.4
76-80	13.3	7.7	23.0
81-90	12.4	7.2	21.6

Median, lower (<5% CI) and upper (>95% CI) values normal range for serum IGF-I assay (Immulite 2500) from Dept. of Biochemistry, University College London Hospital, London, UK. To convert nmol/L to ng/mL divide by 0.131.

Supplementary Table 2. Patient characteristics by GH deficiency status

	All	No GHD	With GHD	P with vs. without GHD [a]
n	39	35	4	
Age at first scan (yrs)	30.5 [24.5, 47.0]	30.4 [24.1, 49.2]	39.4 [29.7, 44.4]	0.50
Range	19.6-66.9	19.6-66.9	27.8-44.7	
Male n (%)	33 (84.6%)	29 (82.9%)	4 (100%)	0.87
Post-menopausal women n (%)	3 (7.7%)	3 (8.6%)	0 (0.0%)	0.70
BMI (kg/m^2)	25.3 [23.1-29.8]	25.0 [21.7-28.6]	29.3 [21.7-28.2]	0.10
Range	17.0-35.0	17.0 -25.0	25.3-33.7	
Moderate-severe TBI	34 (87.2%)	30 (85.7%)	4 (100%)	1.00
Blast TBI n (%)	13 (33.3%)	10 (28.6%)	3 (75.0%)	0.19
Time since TBI (months)	16.3 [3.6, 24.5]	11.4 [3.2, 25.7]	26.8 [7.7-34.3]	0.38
Range	1.5-571.0	1.5-571.0	3.6-34.5	
Time between scans (months)	13.3 [12.1, 14.9]	13.1 [12.1, 14.5]	14.6 [10.3-15.1]	0.58
Range	6.3-24.6	6.3-24.6	9.1-15.1	
Absolute IGF-1 nmol/L [b]	23.8 [19.2-29.5]	24.2 [19.5, 29.5]	19.6 [16.7, 23.2]	0.11
Range	12.8-74.5	12.8-74.5	16.5-23.7	
Age-adjusted IGF-1 ratio	1.1 [0.9, 1.2]	1.1 [0.9, 1.3]	0.9 [0.8-1.0]	0.11
Range	0.6-3.0	0.6-2.9	0.8-1.0	
Any structural brain abnormality n (%)	30 (76.9%)	28 (80.0%)	2 (50.0%)	0.47
≥ 1 contusion	20 (51.3%)	19 (54.3%)	1 (25.0%)	0.56
No. of contusions [c]	1 [0-2]	1 [0-2]	0 [0-0.8]	0.53
Range	0-5	0-5	0-1	
≥ 1 microbleed n (%)	10 (25.6%)	10 (28.6%)	0 (0.0%)	0.53
Superficial siderosis n (%)	13 (33.3%)	11 (31.4%)	2 (50.0%)	0.85
Diffuse axonal injury (DAI) n (%)	5 (12.8%)	4 (11.4%)	1 (25.0%)	1.00

Data given as n (%) or median [IQR] and range (minimum-maximum).

[a] P value for comparison of groups with and without GHD using chi-squared test with Yates' correction for categorical variables and Mann-Whitney U test for continuous variables.

[b] To convert nmol/L to ng/mL divide by 0.131.

[c] including those without any contusions

Supplementary Table 3. Neuropsychological outcome measures in patients after TBI at baseline and recovery over time by IGF-I status.

Symptom domain		Max score	n	First Visit	n	Second visit	Effect of Time [a]	Effect of IGF-I Group [a]	Time*Group Interaction [a]
**Assessment of GH Deficiency in Adults Quality of Life (AGHDA-QoL)**		25	25	9 [2.5-16.5] 0-23	25	7.0 [1-12.5] 0-22	0.99	0.71	0.89
**Beck Depression Inventory II Score (BDI-II)**		63	25	13.0 [4.0-23.0] 0-27	25	11.0 [2.5-18.5] 0-32	0.46	0.75	0.63
**Epworth Daytime Sleepiness Scale**		24	22	3.0 [0.9.5] 0-19	15	4.0 [1.0-10.0] 0-14	0.69	0.45	0.53
**SF-36 QoL**	**Physical functioning**	100	16	70.0 [22.5-98.8] 0-100	24	87.5 [62.3-95.0] 0-100	0.32	0.82	0.53
	**Role limitations due to physical health**	100	17	75.0 [0-100] 0-100	24	50.0 [6.25-100] 0-100	0.58	0.37	0.58
	**Role limitations due to emotional problems**	100	16	100 [66.7-100] 0-100	24	100 [41.7-100] 0-100	0.88	0.59	0.77
	**Energy/Fatigue**	100	16	45.0 [45.0-70.0] 15-85	24	67.5 [47.5-83.8] 5-100	0.41	0.41	0.12
	**Emotional well being**	100	16	70.0 [54-83.0] 28.0-92.0	24	80.0 [61.0-92.0] 24.0-100	0.52	0.75	0.96
	**Social functioning**	100	17	62.5 [50.0-75.0] 12.5-100.0	24	81.3 [50.0-100] 25-100	0.22	0.77	0.87
	**Pain**	100	17	45.0 [39.0-83.8] 22.5-100	24	77.5 [55.6-100] 42.5-100	0.74	0.82	0.93
	**General health**	100	17	60.0 [42.5-80.0] 20-100	24	65.0 [45.0-91.3] 15-100	0.27	0.59	0.18
Cognitive domain	*Cognitive variable*								
**Pre-morbid intelligence: reading ability**	**WTAR raw score**		32	41.0 [35.3-45.0] 0-50	32	42.0 [31.8-46.8] 9-50	0.88	0.83	0.60
**Intellectual ability**	**WASI similarities (verbal)**		33	36.0 [32.5-38.0] 21-46	33	39.0 [35.5-41.0] 23-46	0.61	0.96	0.42
	**WASI matrix reasoning (non-verbal)**		32	26.5 [20.5-30.0] 9-34	32	28.5 [25.3-30.8] 12-35	0.08	1.00	0.20
**Memory: associative memory**	**People test (total score)**		33	22.0 [14.5-29.0] 4-38	33	27.0 [18.0-31.0] 6-38	**0.010**	0.19	0.78
**Memory: immediate recall**	**Logical memory I Total**		33	28.0 [20.5-32.0] 10-40	33	43.0 [33.0-51.5] 19-62	0.26	0.12	**0.046**
	**Logical memory II Total**		33	26.0 [18.5-32.5] 0-42	33	27.0 [19.5-33.0] 10-43	0.63	0.20	0.57
**Memory: delayed recall**	**Logical memory retention**		32	86.7 [78.9-94.3] 33.3-107.7	33	90.3 [82.9-98.0] 45-190	0.41	0.34	0.93
**Processing speed: visual search/complex**	**Trail Making Test Trail A (s)**		32	26.0 [21.0-32.0] 14-67	33	22.0 [16.5-28.5] 13-87	0.82	0.60	0.65
	**Trail Making Test Trail B (s)**		32	52.0 [43.3-68.5] 22-192	33	47.0 [30.5-71.0] 25-166	0.62	0.32	0.81
**Processing speed: naming/reading**	**Stroop Colour Naming (s)**		31	32.0 [28.0-41.0] 20-101	33	32.0 [30.0-35.0] 19-51	0.56	0.90	0.96
	**Stroop Word Reading (s)**		31	23.0 [19.0-32.0] 15-62	32	23.0 [20.0-25.8] 14-43	0.53	0.25	0.34
**Executive function: alternating-switch cost**	**Trail Making Test Trail B minus A (s)**		32	22.5 [13.0-38.8] 4-125	33	19.0 [15.0-42.5] 7-102	0.57	0.24	0.62
**Cognitive flexibility**	**Color Word Stroop Inhibition/switching (s)**		32	66.0 [55.5-76.5] 38-128	33	57.0 [51.5-66.5] 37-119	**0.003**	0.33	0.50
**Word generation fluency**	**DKEFS Letter Fluency F+A+S total**		33	36.0 [27.0-45.0] 11-65	33	41.0 [35.5-48.5] 15-60	0.15	0.57	0.59

[a] corrected for all covariates (time since injury, time between scans, gender, age at first scan, TBI severity)

Data displayed as median, [IQR], range.

Note: AGHDA, BDI-II, Epworth: higher score equals worse symptoms; SF-36: lower score equals worse quality of life; WTAR, Similarities, Matrix reasoning, People test, Logical memory: higher score equals better performance; Trail-making, Stroop, letter fluency: lower score equals better cognition.

Abbreviations: DKEFS, Delis-Kaplan executive function system; (s), seconds; SF-36, Short Form 36 Health Survey; WASI, Wechsler Adult Intelligence Scale; WTAR, Wechsler Test of Adult Reading.
